# Intermittent catheterisation: individuals’ rights, accessibility, and environmental concerns

**DOI:** 10.1038/s41394-024-00651-4

**Published:** 2024-05-31

**Authors:** Andrei Krassioukov, Michel Wyndaele, Matthias Walter, Veronique Keppenne, Blayne Welk, Desiree Vrijens, Francois Theron

**Affiliations:** 1 International Collaboration on Repair Discoveries (ICORD), Faculty of Medicine, The University of British Columbia (UBC), Vancouver, 818 W 10th Ave, V5Z 1M9 BC Canada; 2 GF Strong Rehabilitation Centre, Vancouver Coastal Health Authority, Vancouver, 4255 Laurel St, V5Z 2G9 BC Canada; 3 Division of Physical Medicine and Rehabilitation, Faculty of Medicine, The University of British Columbia (UBC), Vancouver, 4255 Laurel St, V5Z 2G9 BC Canada; 4 Department of Urology, University Medical Center Utrecht, Utrecht, Netherlands; 5 Department of Urology, University Hospital Basel, University of Basel, Basel, Switzerland; 6 Department of Urology, Centre Hospitalier Universitaire (CHU) de Liege, Liege, Belgium; 7 Department of Surgery, and Department of Epidemiology and Biostatistics, Western University, London, ON Canada; 8 Department of Urology, Maastricht University Medical Center, Maastricht, Netherlands; 9 Department of Orthopedics, University of Pretoria, Pretoria, South Africa

**Keywords:** Health care, Quality of life, Public health

## Introduction

Intermittent catheterisation (IC) is the mainstay for bladder management in individuals living with neurogenic lower urinary tract dysfunction (NLUTD), but many are not receiving the best evidence-based standard of IC care available. To garner opinion on individuals’ rights to access IC (including the best available care), representatives from disability organisations (Spinal Cord Injury [SCI] British Columbia, and SCI Ontario, QuadPara Association of South Africa [QASA], and Spina bifida en hydrocephalus [SBH] Nederland) and multidisciplinary experts from the global medical community came together for a ‘meeting of minds’; the aim was to discuss how to improve the lives of individuals living with NLUTD. This article summarises their combined opinion.

NLUTD is a common consequence of neurological disorders, such as SCI, multiple sclerosis, and spina bifida [1], but it is relatively unrecognised by those without personal experience of these conditions. Toileting is a key ‘activity of daily living’ and, therefore, urinary/bowel care is fundamental for independence. NLUTD negatively impacts the health-related quality of life of affected individuals [2], and amplifies the already considerable burden of disability. The countless daily challenges of living with NLUTD – logistical (limited access to adequate bathroom facilities, time burden of IC), emotional (negative feelings associated with distressing events, such as urinary incontinence), psychological (depression, low self-esteem), and social (stigma, social isolation) – limit the lifestyle choices of affected individuals [3–6]. Indeed, the lack of adequate facilities in public bathrooms (limited space, lack of shelves/tables to place equipment, unhygienic areas) in which to perform IC is among the key challenges for IC users [7, 8]. Consequently, such individuals avoid public situations, choosing to stay at home and risk social isolation [7]. NLUTD also has a considerable public economic burden related to the cost of complications, such as urinary tract infections [9, 10], which are more common when suboptimal IC techniques and/or supplies are used.

From the perspective of an individual living with a neurological disorder, NLUTD causes the greatest impairment to daily life and, therefore, maximising bladder control/function is an important goal for recovery. Indeed, restoration of bladder function is a high priority among people with SCI [11]. Unfortunately, these considerations are often overlooked by clinicians who, instead, tend to focus on addressing the underlying neurological disorder.

*‘The lifestyle of a quadriplegic is more impaired by the unintended consequences of disability, such as bladder dysfunction, than it is for people using wheelchairs or assistive devices; our bladder is our* *nemesis.’*

*‘Not having to deal with such consequences sets you free and allows you to live an unhindered lifestyle.’*

**Ari Seirlis, former Chief Executive Officer (CEO), QASA**

Most individuals with NLUTD rely on IC for bladder management. The merits of single-use versus reuse IC are gaining traction [12, 13], and hydrophilic single-use catheters are currently regarded as the optimal choice [13]; furthermore, most catheters on the market have a single-use indication [13, 14]. However, striking geographical differences in the global practices of IC exist, with no identified trends for developing versus developed countries [15], leading to inequality in access to IC for those in need. With the rise in patient empowerment, individuals with disability now act as advocates for change; indeed, users of IC have been pivotal in the drive towards single-use catheters.

*‘If people who use IC get together and refuse to reuse, it sends a strong message; reimbursement bodies are starting to listen.’*

**Ari Seirlis, former CEO, QASA**

## Individuals’ rights and access to intermittent catheterisation

All individuals with NLUTD have the right to access the best evidence-based standard of IC care available and, also have the right to access to adequate and comfortable facilities when performing IC.

*‘It’s a human rights issue. We have shorter lives, and we want a gold standard for bladder management, nothing less.’*

**Ari Seirlis, former CEO, QASA**

**Fig. 1:**
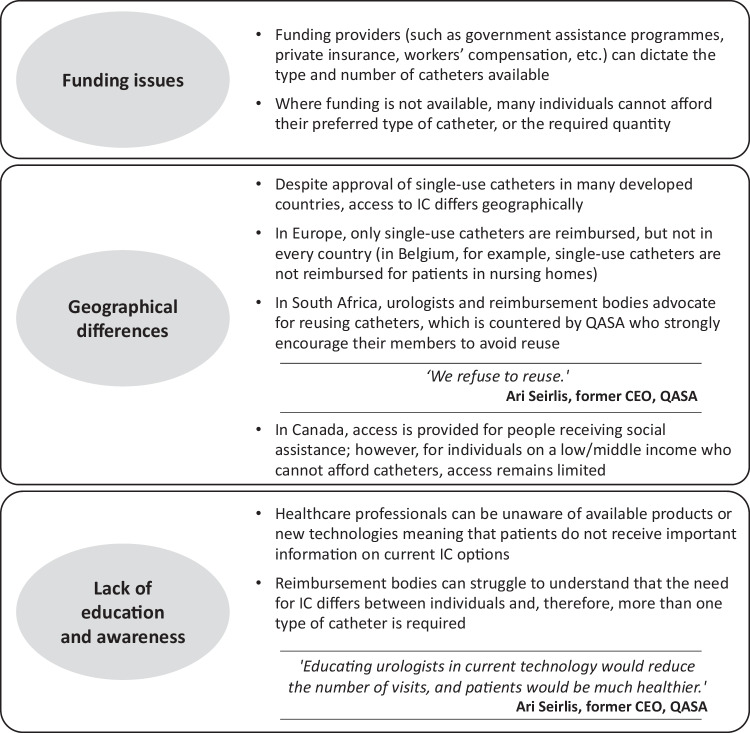
Barriers to accessing the best evidence-based standard of intermittent catheterisation care. *CEO* Chief Executive Officer, *IC* intermittent catheterisation, *QASA* QuadPara Association of South Africa.

Some countries are legally obligated to provide the highest attainable standard of IC care; elsewhere, universal access barriers exist (Fig. 1), which hinder an individual’s freedom to choose the best evidence-based standard of IC.

Using catheters other than the best standard available can have far-reaching consequences. An individual’s lifestyle is restricted due to the challenges of living with NLUTD, and they are constantly reminded of these limitations, from a physical and economic perspective; such issues can be exacerbated by suboptimal IC management. Consequently, there is a negative impact on mental health, which can also be exacerbated by suboptimal IC. In addition, not being able to afford the best standard of IC care may serve as a reminder of poor economic status, which can further impact mental health (through low self-esteem and/or feeling stigmatised, for example).

In countries where resources and funding are limited, reusing catheters is the only option for IC; in other countries, issues with accessibility to single-use IC drives individuals to reuse catheters off-label. However, the long-term physical health effects of continuous reuse are unknown. Regular cleaning of catheters for reuse can result in structural damage [16], which increases the risk of developing urinary tract infections and urethral strictures through urethral trauma [17]. Avoiding the costly health-related consequences of poor bladder management, such as urinary tract infections, could offset the cost of single-use catheters and benefit the whole healthcare system. This is particularly relevant for developing countries where reimbursing single-use catheters could reduce the demand on overstretched resources that are used to treat urinary tract infections resulting from poor bladder management.

*‘The consequences are massive when moving away from a gold standard of treatment.’*

**Peter Athanasopoulos, SCI Ontario**

## Improving access to intermittent catheterisation

Article 25 of the ‘Convention on the rights of persons with disabilities’ 2006, states that ‘…persons with disabilities have the right to the enjoyment of the highest attainable standard of health without discrimination on the basis of disability’ [18]. To avoid violating the human rights of people living with NLUTD, the best standard of IC care must not become an option accessible only to wealthy individuals and developed countries. One solution is not enough – in countries where single-use catheters are available, authorities have a moral and ethical responsibility to provide such care as the minimum requirement, regardless of income. Where reuse is the only option, industry has a similar responsibility to create reusable solutions that provide optimal bladder management with minimal negative impact on safety.

Education and raising awareness of IC and available catheter options is vital and can be achieved in several ways.

1. **Peer-to-peer exchange –** international medical societies and organisations should strive to educate specialists through provision of up-to-date information on current research and literature relating to available products and new technologies.
2. **Promotion of an interdisciplinary approach –** enables a group of healthcare professionals with various areas of expertise to collaborate with reimbursement bodies to acknowledge all aspects of the challenges faced by individuals with neurological disorders, including bladder management.
3. **Education of specialist nurses –** often, specialist nurses have most contact with people living with neurological disorders and NLUTD; investing in well-informed specialist nurses positively impacts time, resource, and cost.

To improve access, it is imperative for gatekeepers, such as medical insurance companies and governmental health authorities, to acknowledge the IC need of individuals with NLUTD. It is also essential that urologists and rehabilitation doctors highlight to gatekeepers the importance of single-use catheters in improving the daily lives of these individuals.

## The environmental agenda versus individuals’ rights

Previously, we discussed the issues of sustainability with disposable catheters in the context of the health and safety of IC users [13]. People may actively choose to reuse catheters, not only to reduce the environmental impact of catheter use, but also to avoid the stigma of using excessive amounts of plastic. However, it is important to reiterate the risk of jeopardising health when reusing catheters, since the effects of long-term reuse are unknown. The perception among disability organisations is that the environmental agenda is prioritised over the health of people living with neurological disorders.

*‘We are at the bottom of the food chain when it comes to these discussions. The environmental agenda gets more attention than the health and well-being of individuals, which we resent.’*

**Ari Seirlis, former CEO, QASA**

Industry has a responsibility to reduce the environmental impact of single-use catheters for IC. Possible approaches include implementing a system for collecting and recycling used products and conducting research into recyclable and biodegradable materials [13].

*‘There is a desire among members for industry to innovate.’*

**Chris McBride, SCI BC**

Acknowledging that reuse is the only option in some countries, the need to reduce single-use plastics must not be used as a smokescreen to promote the off-label reuse of catheters, simply to save money in countries where optimal care is available. The health of individuals in need of IC is more important than the financial gains associated with reuse and the environmental agenda per se.

## Conclusions

The protection of human rights for people living with neurological disorders and NLUTD is an ethical and moral imperative and is of crucial importance if these individuals are to live healthy lives. First and foremost, everyone in need of IC has a right to access catheters and, unless national/local resources and funding are extremely limited, there is no excuse not to provide the best standard of IC care (i.e., hydrophilic single-use catheters). Fundamentally, access to IC is at the mercy of reimbursement bodies who, often, are far removed from the medical community and do not understand the complex heterogeneous needs of individuals living with NLUTD. By improving awareness and understanding of individuals’ rights, we can address the barriers limiting access to catheters and enhance the IC landscape for people living with NLUTD. Until then, the medical community and IC users must deliver a united front to make sure the voice of the individual is heard and to advocate for the best options for standard of IC care.

*‘This work is critical to our well-being and will improve the lives of those to come.’*

**Ari Seirlis, former CEO, QASA**

## Data Availability

Data sharing not applicable as no datasets were generated and/or analysed for this study.
